# Low irisin levels are associated with increased body weight and an adverse metabolic profile

**DOI:** 10.20945/2359-4292-2024-0441

**Published:** 2025-06-30

**Authors:** Milene Moehlecke, Jakeline Rheinheimer, Daisy Crispim, Manoel Roberto Maciel Trindade, Cristiane Bauermann Leitão

**Affiliations:** 1 Programa de Graduação em Ciências Médicas: Endocrinologia, Univesidade Federal do Rio Grande do Sul, Porto Alegre, Rio Grande do Sul, Brazil; 2 Serviço de Endocrinologia, Hospital de Clínicas de Porto Alegre, Porto Alegre, RS, Brazil; 3 Serviço de Cirurgia do Aparelho Digestivo, Hospital de Clínicas de Porto Alegre, Porto Alegre, RS, Brazil

**Keywords:** Body mass index, Obesity, Metabolic diseases, Diabetes mellitus, type 2

## Abstract

**Objective:**

To assess whether plasma irisin is associated with anthropometric, body composition, and metabolic parameters according to body mass index.

**Methods:**

A prospective cohort study was conducted at a tertiary referral hospital in Southern Brazil. Patients were divided according to body mass index: Group 1 (body mass index = 18.5 - 29.9 kg/m2; n = 15), Group 2 (body mass index = 30 - 39.9 kg/m2; n = 36), and Group 3 (body mass index ≥ 40 kg/m2; n = 30). Groups 1 and 2 underwent cholecystectomy, while Group 3 underwent Roux-en-Y gastric bypass. All groups were evaluated at baseline, and Groups 2 and 3 were re-evaluated 6 months later. Body composition was assessed using X-ray absorptiometry, and resting energy expenditure was measured by indirect calorimetry.

**Results:**

The study subjects were predominantly female (75%), white (82%), with a mean age of 46 ± 14 years. Group 3 had lower irisin levels compared to Group 2: 9.1 ± 2.2 *versus* 10.7 ± 2.8 ng/mL; p = 0.038). Irisin was negatively correlated with weight (r = -0.246; p = 0.042), waist circumference (r = -0.272; p = 0.024), glucose (r = -0.259; p = 0.039), glycated hemoglobin (r = -0.283; p = 0.024), triglycerides (r = -0.414; p = 0.024), and positively correlated with HDL (r = 0.280; p = 0.029).

**Conclusion:**

Irisin showed an inverse correlation with insulin-related metabolic pathways, suggesting its potential involvement in insulin resistance states such as obesity and type 2 diabetes.

## INTRODUCTION

Obesity, defined as a body mass index (BMI) equal to 30 kg/m^2^ or higher, results from a chronic imbalance between energy intake and expenditure (^1^). Peripheral signals from the gastrointestinal tract, adipose tissue, and muscle interact with the central nervous system to ultimately determine body weight (^2^). Skeletal muscle and adipose tissue are increasingly recognized as endocrine organs that release a variety of molecules, termed myokines and adipokines, respectively. These molecules have been proven to play roles in body weight regulation (^3^).

Two types of adipose tissue are described in mammals: white adipose tissue (WAT), functioning as the dominant site for lipid storage, and brown adipose tissue (BAT), which is the site of thermogenesis through uncoupled respiration (^4^). The main function of BAT is the maintenance of temperature homeostasis. Until recently, BAT was demonstrated in human newborns but not in adults. However, metabolically active BAT has now been identified in healthy adult humans (^5^). It appears to have a role in body weight regulation, as its activity is negatively correlated with body fat and BMI, and positively with energy expenditure (^6^).

In early 2012, Boström and cols. (^7^) discovered an exercise-related plasmatic myokine named irisin. Exercise stimulates the transcription of peroxisome proliferator-activated receptor-γ coactivator 1〈 (*PGC-1*〈), which leads to the shedding of fibronectin type III domain-containing 5 (FNDC5) into the circulation (^8^). FNDC5 is then cleaved, and the metabolically active hormone irisin is released into the bloodstream. Irisin acts on white adipocyte precursors (^9^) and promotes a process known as “browning” of WAT (^10^). This process results in the formation of beige, or “brite”, adipocytes, which share some characteristics with BAT, such as an increased number of mitochondria. Beige adipocytes are identified by their increased expression of uncoupling protein 1 (*UCP1*), a traditional marker of BAT, which participates in energy metabolism by enhancing thermogenesis (^11^). In a rodent model, an increase in irisin was associated with a reduction in body weight and improvements in metabolic parameters, such as glucose metabolism (^7^).

Since its initial discovery, irisin has attracted interest as a potential strategy to combat obesity and its associated disorders in humans. Studies have investigated the relationship between irisin levels BMI (^12^), body composition, insulin sensitivity (^13^), metabolic syndrome (^14^), and non-alcoholic liver disease (^15^). Higher circulating levels of irisin are associated with increased insulin levels, improvements in glucose homeostasis, and lower triglyceride levels (^13^,^15^). These findings suggest that irisin may also play a role in intermediary metabolism in humans. However, the precise effects of irisin on body composition, glycemic and lipid metabolism remain to be elucidated.

Given this context, this study aimed to assess whether plasma irisin is associated with anthropometric, body composition, and metabolic parameters according to body mass index. Additionally, we analyzed plasma irisin levels following surgically induced massive weight loss in comparison to obese controls. Lastly, we examined whether polymorphisms in key genes related to the irisin system (rs8192678 in the *PGC-1*〈 gene, rs3480 in the *FNDC5* gene, and rs3826 in the *UCP1* gene) were associated with anthropometric, body composition, and metabolic parameters, as well as *UCP1* gene expression in adipose tissue.

## METHODS

### Subject characteristics

This prospective cohort study involved three groups of patients categorized by BMI. Group 1 consisted of patients who were eutrophic or overweight, with a BMI between 18.5 and 29.9 kg/m^2^ (n = 15), Group 2 had patients with a BMI between 30 and 39.9 kg/m^2^ (n = 36), and Group 3 comprised patients with a BMI over 40 kg/m^2^ (n = 30). Groups 1 and 2 patients underwent cholecystectomy, and Group 3 underwent open gastroplasty with Roux-en-Y gastric bypass. The groups were assessed at baseline, with Groups 2 and 3 reevaluated 6 months post-surgery. Recruitment was conducted at a tertiary referral hospital, following institutional review board approval and after obtaining written informed consent from all participants.

Eligibility was restricted to individuals aged 18 and above. Exclusion criteria included impaired thyroid function, cancer, current systemic corticosteroid treatment, acute or chronic viral hepatitis, any acute inflammatory disease, or usage of medication known to affect energy expenditure. Clinical and anthropome-tric measurements, body composition, resting energy expenditure (REE), biochemical profiles, and physical activity levels were evaluated preoperatively for all subjects and 6 months postoperatively for Groups 2 and 3. Additionally, *UCP1* gene expression in adipose tissue was analyzed at baseline. The study is registered under the Certificate of Presentation for Ethical Consideration (CAAE no. 09054812.8.0000.5327).

### Clinical and anthropometric measurements

During face-to-face interviews, standard questionnaires gathered socio-demographic data, including alcohol consumption, smoking status, and lifestyle habits, such as physical activity, health history, and medication use. Individuals who had smoked regularly in the past year were identified as active smokers. Systemic arterial hypertension was defined as blood pressure ≥ 140/90 mmHg measured on two occasions or the current use of antihypertensive medication. Anthropometric measurements comprised body weight (in kg) and height (in meters) with the patients in light clothing and barefoot, waist circumference (in cm, measured at the level of the umbilical scar), and BMI (calculated as the weight/height^2^ ratio). Physical activity level was quantified using a pedometer (Yamax Digi-Walker SW-700, Japan), individually calibrated for each patient, and evaluated according to the mean of steps during 8 consecutive days, 1 month before and 6 months after surgery.

### Body composition and resting energy expenditure measurements

Body composition, including fat mass and fat-free mass (FFM), was determined using dual-energy X-ray absorptiometry (DXA; Lunar Prodigy Advance, GE Medical Systems, USA). Resting energy expenditure was measured by open-circuit indirect calorimetry (Model 7100, Korr Medical Technologies^®^, USA), with respiratory data collected over a 30-minute period in the morning after a 12-hour fast.

### Biochemical and hormonal parameters

Blood samples were collected before the surgery and 6 months after. Fasting plasma glucose levels were measured using the glucose-oxidase method, and glycated hemoglobin (HbA1c) was assessed through high-performance liquid chromatography (Merck-Hitachi 9100, Merck, Germany); the reference values were 4 to 6%. The American Diabetes Association’s (ADA) criteria established the diagnosis of type 2 diabetes (T2D) and prediabetes (^16^). Triglyceride and cholesterol levels were determined via enzymatic methods, whereas low-density lipoprotein (LDL) cholesterol was computed using the Friedewald equation. Serum insulin levels were quantified by radioimmunoassay (Modular Analytics E170, Roche Diagnostics, Germany), and insulin resistance was estimated with the homeostasis model assessment (HOMA-IR = [fasting insulin (mUI/mL) x fasting plasma glucose (mmol/L)]/22.5) (^17^).

Plasma irisin levels at baseline and 6 months later were measured using a competitive ELISA kit IX-067-29 (Phoenix Pharmaceuticals, USA), as described elsewhere (^18^). The linear range of the standard was 1.24 to 20.0 ng/mL, with intraand inter-assay coefficients of variability reported by the manufacturer as less than 10% and 15%, respectively. The minimum detectable concentration was established at 1.24 ng/mL. Plasma samples were coded, and assays conducted in duplicate by one researcher to ensure consistency.

### Quantification of *UCP1* gene expression by real-time polymerase chain reaction

Subcutaneous abdominal adipose tissue biopsy (100 mg) was collected from patients during elective surgery. The samples were immediately stored in RNALater (Thermo Scientific, USA) at -80 °C until analysis. Total RNA was extracted using the first step of the MirVana miRNA Isolation Kit (Thermo Scientific, USA), with concentration and quality assessed on a NANODROP 2000 spectrophotometer (Thermo Scientific, USA). Only RNA samples with purity ratios (A260/A280 = 1.9 - 2.1) were used in subsequent analyses (^20^). The mean RNA concentration ± standard deviation (SD) from the adipose tissue was 80 ± 31 ng / 100 mg. Real-time reverse transcription of polymerase chain reaction (RT-qPCR) was conducted in two separate reactions: first, RNA was reverse transcribed into cDNA, then cDNA was amplified by quantitative RT-PCR (RT-qPCR). Reverse transcription of 250 ng of RNA into cDNA was carried out using the SuperScript VILO Master Mix for RT-PCR (Thermo Scientific, USA) following the manufacturer’s protocol for the random primer method.

Quantitative real-time reverse transcription of polymerase chain reaction experiments were performed in a 7500 Fast Real-Time PCR System Thermal Cycler (Thermo Scientific, USA). Experiments were performed by monitoring in real-time the increase in fluorescence of the SYBR Green dye. Primers for *UCP1* were designed from published sequences using Primer Express 3.0 Software (Thermo Scientific, USA), with sequences being *UCP1* Forward 5’-GCCATCTCCACGGAATCAAA-3′, *UCP1* Reverse 5′CCTTTCCAAAGACCCGTCAAG-3′. Each sample was assayed in triplicate and a negative control was included in each experiment. Quantitative real-time reverse transcription of polymerase chain reaction specificity determined using melting curve analyses and the primers generated amplicons that produced a single sharp peak during the analyses (^19^). Quantification of *UCP1* was performed by relative quantification using the comparative ∆?C∆C_q_ method (^20^) and expressed relative to the reference gene (β*2M*), which is used to calculate changes in gene expression changes as fold difference between experimental and external calibrator samples (^20^).

### Genotyping

For genotyping, DNA was extracted from peripheral blood leukocytes using the salting-out method. The rs8192678 (G/A) polymorphism in the *PGC1-*〈 gene, rs3460 (A/G) in the *FNDC5* gene, and rs1800592 (A/G) in the *UCP1* gene were determined by real-time PCR using the Human Custom TaqMan Genotyping Assay (Thermo Scientific, USA). All reactions occurred in 384-well plates on the ViiA 7 Real-Time PCR System (Thermo Scientific, USA), as outlined in a previous study by our group (^21^).

### Statistical analysis

Our sample size was calculated to achieve 80% power (〈 = 5%), taking into account previously published values of circulating irisin’s SD (^19^). Consequently, the sample size was set at a minimum of 17 volunteers per group to detect differences of at least 1 SD in irisin levels among groups. The normality of variable distribution was assessed using the Kolmogorov-Smirnov test. Quantitative data are presented as mean and SD or median and interquartile range, depending on the distribution of the variables. Variables with non-normal distribution were log transformed. Categorical data are shown as frequencies, and their differences were analyzed using the Chi-squared or Fisher’s exact test.

Correlations between continuous variables were evaluated using Pearson’s or Spearman’s correlation coefficients. Multiple linear regression analyses were conducted with plasma irisin levels as the dependent variable. Multivariate logistic regression analysis was applied to examine the association between irisin levels and the presence of prediabetes or T2D. The generalized estimating equation was utilized to assess differences in irisin levels between groups at follow-up, adjusted for age, sex, and physical activity level. Furthermore, the generalized estimating equation was used to study the effects of the weight loss following bariatric surgery on irisin levels in patients with class 3 obesity (Group 3) compared to controls class 1 and 2 obesity (Group 2), after adjustments for age, body composition components, BMI, waist circumference, and step count. For analyzing results, REE was estimated, adjusting for the patient’s physical activity level using a pedometer. Analysis of variance (ANOVA) with Tukey’s *post-hoc* test was performed for multiple pairwise comparisons. Data were analyzed using Statistical Package for the Social Sciences (SPSS), version 21.0 (IBM SPSS Statistics, USA), and p < 0.05 (two-tailed) was considered significant.

## RESULTS

### Patient’s characteristics at baseline

Eighty-one patients were analyzed, most of whom were female (75%), white (82%), and had a mean age of 46 ± 14 years. Baseline demographic, anthropometric, metabolic, and hormonal parameters according to BMI categories are presented in **Table 1**. Twenty-one patients (26%) had T2D, and 38 (47%) had prediabetes, with no significant difference among groups (p = 0.162 and 0.841, respectively). No differences were observed among groups regarding smoking status (p = 0.338) or physical activity level (p = 0.091). Irisin levels did not differ according to sex (p = 0.643). As expected, all anthropometric and body composition components increased with rising BMI. Resting energy expenditure also increased with higher BMIs (1,460 ± 402 *versus* 1,842 ± 581 *versus* 2,202 ± 615 kcal/day; p < 0.001), but no differences among groups were observed when adjusted for FFM (32 ± 6 *versus* 36 ± 18 *versus* 33 ± 8 kcal/kg of FFM; p = 0.629).

**Table 1:** Baseline characteristics according to body mass index

	Group 1 (n = 15)	Group 2 (n = 36)	Group 3 (n = 30)	p-value^*^
BMI, kg/m^2^	(18.5-29.9)	(30-39.9)	(≥ 40)	
Demographic characteristics				
Age, years	51 ± 21	47 ± 13	43 ± 11	0.161
Gender, female/male	7/4	28/8	25/5	0.403
Ethnicity, white	7 (64)	30 (83)	26 (87)	0.218
Non-smokers	8 (73)	26 (72)	16 (53)	0.494
Type 2 diabetes	2 (13.3)	8 (22.2)	11 (36.7)	0.162
Steps walked/day	3,331 (1,966 - 10,783)	4,341 (1,160 - 9,751)	5,649 (1,179 - 28,702)	0.091
Anthropometric characteristics				
Weight, kg	63 ± 11	90 ± 15†‡	128 ± 18§	<0.001
BMI, kg/m^2^	24 ± 19	34 ± 5†‡	48 ± 9§	<0.001
Waist circumference, cm	79 ± 12	100 ± 10†‡	124 ± 14§	<0.001
Body composition				
Fat mass, kg	21 ± 1	37 ± 10†‡	64 ± 14§	<0.001
Fat mass, %	34 ± 2.0	43 ± 1.0	51 ± 1.1	<0.001
Fat-free mass, kg	43 ± 9	54 ± 13‡	67 ± 20§	<0.001
Energy expenditure				
REE, kcal	1,338 ± 170	1,842 ± 94†‡	2,202 ± 115	<0.001
REE/weight, kcal/kg	21 ± 4	20 ± 5†	17 ± 5	0.027
REE/fat-free mass, kcal/kg	32 ± 6	36 ± 18	33 ± 8	0.629
Metabolic parameters				
Fasting glucose, mg/dl	121 ± 29	125 ± 33	144 ± 55	0.167
Glycated hemoglobin, %	5.3 ± 0.7	5.8 ± 0.9	6.4 ± 1.3§	0.032
Total cholesterol, mg/dL	165 ± 42	185 ± 49	181 ± 34	0.503
LDL cholesterol, mg/dL	153 ± 42	164 ± 46	146 ± 35	0.261
HDL cholesterol, mg/dL	46 ± 15	42 ± 9	38 ± 10	0.149
Triglycerides, mg/dL	50 (18 - 123)	91 (29 - 341)	131 (67 - 382)‡§	<0.001
Hormonal parameters				
Fasting insulin, µIU/mL	19 (17)	39 (23)	44 (36)	0.177
HOMA-IR	2.2 (1.1 - 3.4)	11.1 (5.8 - 19.5)†	12 (7.2 - 20.4)§	0.014
Plasma irisin, ng/mL	8.8 ± 3.0	10.7 ± 2.8	9.1 ± 2.2‡	0.038

Results presented as means ± standard deviation, proportion, n (%) or median (interquartile range).

BMI: body mass index; REE: resting energy expenditure; LDL: low-density lipoprotein; HDL: high-density lipoprotein; HOMA-IR: homeostatic model assessment of insulin resistance.

* ANOVA test with Tukey *post hoc*; † p-value < 0.05 for Group 1 *versus* Group 2; ‡ p < 0.05 for Group 2 *versus* Group 3; § p < 0.05 for Group 1 *versus* Group 3.

### Irisin association with anthropometric and body composition measurements

**Figure 1:**
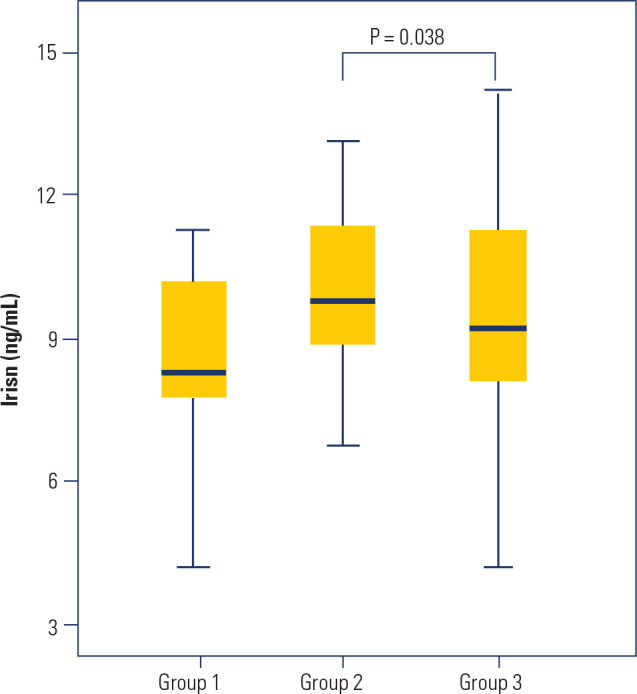
Plasma irisin levels according to the body mass index categories at baseline. Group 1: body mass index of 18.5 to 29.9 kg/m^2^; Group 2: body mass index of 30.0 to 39.9 kg/m2; Group 3: body mass index of ≥ 40.0 kg/m^2^. p for analysis of variance *post-hoc* comparisons: Group 1 *versus* Group 2: p = 0.850; Group 1 *versus* Group 3: p = 1.000; Group 2 *versus* Group 3: p = 0.038.

Plasma irisin levels according to BMI categories are depicted in **Figure 1**. Group 2 exhibited higher irisin levels compared to Group 3: 10.7 ± 2.8 *versus* 9.1 ± 2.2 ng/mL, respectively; p = 0.038. Irisin levels inversely correlated with body weight (r = -0.246; p = 0.042) and waist circumference (r = -0.290; p = 0.016). No significant correlations were found between irisin levels and BMI (r = -0.214; p = 0.078), FFM (r = -0.195; p = 0.116), or fat mass (r = -0.172; p = 0.204).

HDL: high-density lipoprotein.

Fifty-five patients were reevaluated 6 months post-surgery. Eleven patients who were not reevaluated belonged to Group 2, with the reasons including difficulty in contacting them (n = 9), pregnancy (n = 1), and one relocating to another city (n = 1). Thirty patients from Group 3 underwent Roux-en-Y gastric bypass, with their percentage of excess weight loss being 54 ± 12% at follow-up. The percentage of body weight lost as fat mass was 45%, and as FFM was 24%. Body mass index remained stable in Group 2 (34 ± 4 *versus* 34 ± 3 kg/m^2^; p = 0.530) during follow-up. Anthropometric, body composition variables, and REE at follow-up are described in **Table 2**. The physical activity level at 6 months, measured as median step count/day, was 3,465 (2,627-5,868) for Group 2 and 4,510 (3,767-8,065) for Group 3, with no significant difference compared to preoperative values.

**Table 2:** Anthropometric, body composition parameters, and resting energy expenditure in the Groups 2 and 3, at 6 months

	Group 2 (n = 25)	Group 3 (n = 30)	p-value^*^
Weight, kg	91 ± 3	97 ± 3	0.072
Body index mass, kg/m^2^	32 ± 30	35 ± 32	1.000
Waist circumference, cm	104 ± 46	123 ± 46	0.103
Fat-free mass, kg	53 ± 68	59 ± 74	1.000
Fat mass, kg	33 ± 58	34 ± 58	1.000
Fat mass, %	41 ± 27	37 ± 27	0.173
Resting energy expenditure, kcal/day	1,971 ± 2,252	1,831 ± 2,263	0.431

* Generalized estimating equation and Bonferroni *post-hoc*.

All variables were quite similar between groups at follow-up, including irisin levels in Group 2 (8.8 ± 0.6) and Group 3 (8.1 ± 0.5 ng/mL), p = 0.515, despite a decrease observed in both groups (p < 0.001). To analyze whether there was a difference in variables between the groups over time, we also calculated the delta of the means after 6 months (**Supplementary table 2**). Glucose levels significantly improved 6 months after surgery compared to baseline: 90.5 ± 17 *versus* 144 ± 55 mg/dL (p = 0.011), respectively. Following surgery, only 3 of 11 patients met the criteria for T2D.

### Low irisin levels are associated with a poor metabolic profile

Circulating irisin levels correlate negatively with serum glucose (r = -0.265; p = 0.033), HbA1c (r = -0.303 p = 0.015), and serum triglycerides (r = -0.451 p < 0.001), and positively with HDL (r = 0.307; p = 0.015). No significant correlations were observed between plasma irisin and total cholesterol (p = 0.714) or HOMA-IR (p = 0.438).

**Figure 2:**
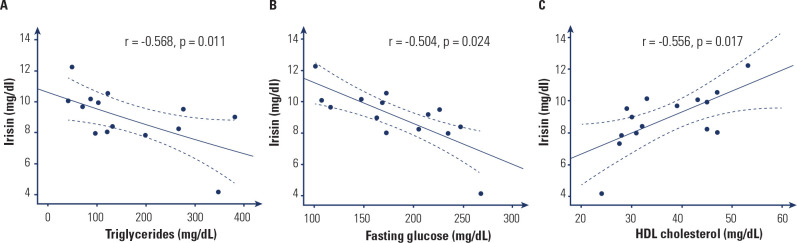
Correlations between circulating irisin concentrations and triglycerides (**A**), fasting glucose **(B**) and high-density lipoprotein cholesterol (**C**), patients with type 2 diabetes. Values for r and p are indicated in each graph. Dotted lines indicate the 95% confidence interval of the regression.

Separate multivariable models, with HbA1c, triglycerides, and HDL as the dependent variables and irisin as the independent variable, were adjusted for age, waist circumference, and physical activity (in steps/day) (**Table 3**). The associations between plasma irisin and HbA1c, triglycerides, and HDL remained significant after these adjustments. Alternative models substituting waist circumference with body weight yielded similar results. Among patients with T2D, irisin levels correlated negatively with glucose (r = -0.504; p = 0.024), and triglycerides (r = -0.568; p = 0.011) and positively with HDL (r = 0.556; p = 0.017) (**Figure 2**).

**Table 3:** Multivariate regression analysis for circulating irisin levels

Independent variables	HbA1c		Tryglicerides		HDL	
	â	p-value	â	p-value	â	p-value
Irisin, ng/mL	-0.208	0.023	-0.990	0.003	25	0.049
Age, years	0.166	0.021	0.005	0.982	11.88	0.211
WC, cm	0.001	0.023	0.006	0.001	-0.141	0.055
Physical activity, steps/day	-0.016	0.643	0.025	0.843	2.48	0.616

HbA1c: glycated hemoglobin; HDL: high-density lipoprotein; WC: waist circumference.

Separate multivariable models were built with HbA1c, triglycerides and HDL as the dependent variables, and irisin as the independent variable, adjusted for age, waist circumference, and physical activity (in steps/day)

### *UCP1* gene expression in adipose tissue was not associated with clinical or laboratory variables

*UCP1* gene expression showed no correlation with clinical parameters such as weight, BMI, blood pressure, and physical activity, nor with laboratory or hormonal variables, including plasma irisin levels (data not shown).

### Clinical and laboratory features of patients, according to the presence of the risk alleles of *PGC-1*〈 rs8192678, *FNDC5* rs3480, and *UCP1* rs3826 polymorphisms

Clinical and laboratory features of patients according to the presence of the minor alleles of each analyzed polymorphisms were described in **Supplementary table 1**. The evaluated genotypes were in Hardy-Weinberg equilibrium (p > 0.05). Patients with the *PGC-1*〈 rs8192678 A allele showed higher weight (106.4 ± 28.0 *versus* 91.4 ± 29.0 kg; p = 0.035), waist circumference (117.9 ± 20.4 *versus* 107.5 ± 21.9 cm; *p* = 0.048), and lower HDL cholesterol levels (37.7 ± 7.8 *versus* 45.4 ± 12.1 mg/dL; p = 0.004) than G/G carriers, respectively. The G allele of *FNDC5* rs3480 was not linked to any adverse clinical or laboratory characteristics. Conversely, patients with the G allele of *UCP1* rs3826 demonstrated increased weight (105.4 ± 31.6 *versus* 91.7 ± 22.5 kg; p = 0.037), FFM (61.9 ± 21.0 *versus* 50.9 ± 12.6 kg; p = 0.019), and HbA1c (6.3 ± 1.3 *versus* 5.7 ± 0.7%; p = 0.028), compared to A/A carriers. Irisin levels did not correlate with the expression of the risk alleles of PGC-1〈 rs8192678 (r = -0.096; p = 0.451), FNDC5 rs3480 (r = -0.126; p = 0.321), and UCP1 rs3826 polymorphisms (r = -0.065; p = 0.606).

## DISCUSSION

Our data indicated an increase in circulating irisin le-vels in patients with class 1 and 2 obesity (Group 2) but not in those with class 3 obesity (Group 3). Additionally, irisin levels were inversely correlated with anthropometric measurements (body weight and waist circumference) and metabolic parameters (plasma glucose, HbA1c, and triglycerides). We also observed a reduction in irisin levels over time in both groups of patients with obesity.

To date, few prospective studies have evaluated irisin behavior following weight loss interventions over a period of up to 1 year (^18^,^22^-^24^). Most of them have shown unchanged or decreased irisin levels during this period. One hypothesis for this finding, despite the clear metabolic improvements from surgery-induced weight loss, is the concomitant loss of muscle mass. Huh and cols. reported that decreased irisin levels after surgically induced weight loss, paralleling a reduction in body weight over a 6-month follow-up (^19^). Interestingly, when the decrease in circulating irisin levels was adjusted to FFM, the statistical significance was lost. In our study, adjustments for FFM showed no differences in irisin levels at baseline and 6 months later. Similarly, Lee et al. (^23^) found a significant correlation between preoperative irisin concentration and the percentage of body weight lost after one year in patients with class 3 obesity.

In contrast, Demirpence and cols. observed no significant changes in irisin levels 3 months post-bariatric surgery (^23^), and Majorczyk and cols. (^24^) found no change in irisin levels 6 months and 1 year post-surgery in their prospective study of 40 patients treated for morbid obesity. Nonetheless, none of these studies included body composition analyses following bariatric surgery.

In humans, irisin levels appear to increase according to obesity class (^18^,^25^). Our data shows a numerical increase in irisin levels between individuals of normal weight and those with class 1 and 2 obesity. Huh and cols. (^18^) speculated that this might represent a compensatory mechanism. Nonetheless, among individuals with class 3 obesity, we observed a reduction in irisin levels compared to Group 2, albeit levels remained numerically higher than those in individuals of normal weight. Although initially described as a protective factor against weight gain through increased energy expenditure via browning of the WAT subtype, it seems that in the class 3 obesity population, lower irisin levels might reflect a failure in activation in response to weight gain. Cypess and cols. (^5^) identified regions of functionally active BAT in adult humans, with a decrease in volume correlating with increased BMI. Similarly, van Marken Lichtenbelt and cols. (^26^) showed a negative linear relationship between BAT activity, BMI, and body fat percentage.

Thus, irisin might also play a role in compensatory mechanisms for metabolic regulation in unhealthy patients, potentially failing in extreme conditions such as class 3 obesity and T2D. Yan and cols. (^14^) described an inverse correlation between circulating irisin and components of the metabolic syndrome similar to our results. Choi and cols. (^27^) described reduced irisin levels in newly diagnosed T2D patients and a lower odds ratio for T2D with increased plasma irisin levels. Another study indicated a continuous risk for T2D with progressively lower irisin levels among individuals with normal glucose tolerance or prediabetes (^28^). These findings align with our findings.

In our study, the *PGC-1*〈 rs8192678 A allele was associated with increased weight, waist circumference, and reduced HDL cholesterol but was not correlated to BMI. One hypothesis for this finding is that the increased weight may be linked to a greater amount of visceral fat rather than total fat. Another possibility is that our sample size was too small to identify a statistically significant difference in BMI. Additionally, the *UCP1* rs3826 G allele was associated with higher weight, higher FFM, and HbA1c levels. Our group has previously identified an association between two polymorphisms in the gene encoding irisin, *FNDC5*, and increased HbA1c, higher systolic blood pressure, and dyslipidemia in women with T2D (^29^). Reduced expression and activity of *PGC1-*〈, a co-factor that stimulates *FNDC5* and, by extension, irisin release, have been reported in patients with T2D (^30^). Given that *PGC1-*〈 is a crucial regulator of mitochondrial homeostasis, its diminished function may contribute to insulin resistance in conditions such as obesity and T2D (^31^).

Our results must be interpreted with caution due to several limitations. Firstly, our findings on plasma irisin levels and anthropometric and metabolic parameters are based on an observational study subject to confounding factors. This limitation, however, does not diminish the value of this study because we were able to adjustments for numerous potential confounders, including physical activity, which may influence plasma irisin levels. Another limitation is the relatively small sample size of eutrophic subjects, potentially responsible for some observed negative results.

In summary, circulating irisin levels increase in individuals with class 1 and 2 obesity, but not in those with class 3 obesity, who exhibit a failure in irisin activation in response to weight gain. Moreover, irisin levels were significantly reduced 6 months post-surgery. Whether the decrease in circulating irisin levels is attributable to a reduction in muscle mass or a direct effect of surgically induced weight loss remains unclear. Additionally, irisin levels demonstrated an inverse correlation with insulin-related metabolic pathways, suggesting a potential role for irisin in insulin resistance states, such as obesity and T2D. The regulation and specific role of irisin in anthropometric and metabolic parameters in humans, both in health and disease, require further clarification. Our observations suggest that irisin may play a role in body weight regulation and insulin sensitivity in humans.

## Data Availability

the authors confirm that this work is original, and neither the manuscript nor any parts of it, except for abstracts of less than 250 words, have been published previously or are under consideration for publication elsewhere.

## SUPPLEMENTARY MATERIAL

**Supplementary table 1:** Primers and probes

SNPs	Primers	Probes
rs3480 (FNDC5)	F 5’AGCTCTTGTAGACCGGAAGGAA3’	FAM-5’CCATCACCCAATGAC3’
	R 5’TGGTCCCCAAGCCAGAGA3’	VIC -5’AGCCATCACCTAATGAC3’
rs8192678 (PGC1-〈)	F 5’GGTCATCCCAGTCAAGCTGTTTT3’	FAM-5’ CAGTTCACCGGTCTTG3’
	R 5’TGGAGAATTGTTCATTACTGAAATCACTGT3’	VIC-‘5 CTCAGTTCACTGGTCTTG3’
rs1800592 (UCP1)	R 5’CTCCTTTCCTTTTTGAAAATGTAGAACACA3’	FAM- ‘AATGCACTCGATCAAA’3
	F 5’TTTGTGCAGCGATTTCTGATTGAC3’	VIC- ‘5CAAATGCACTTGATCAAA3’

SNP: single nucleotide polymorphism.

**Supplementary table 2:** Anthropometric, body composition parameters, and resting energy expenditure according to the mean delta between Groups 2 and 3 at 6 months

	∆ Group 2	∆ Group 3	p-value^*^
Weight, kg	0.4 ± 2.5	33 ± 11	< 0.001
Body index mass, kg/m^2^	0.1 ± 0.9	13 ± 4	< 0.001
Waist circumference, cm	0.1 ± 3.4	21 ± 10	< 0.001
Fat-free mass, kg	8.5 ± 21.2	27 ± 25	< 0.001
Fat mass, kg	-0.1 ± 1.9	23 ± 8	< 0.001
Fat mass, %	-0.8 ± 1.5	9 ± 5	< 0.001
Resting energy expenditure, kcal/day	-5.7 ± 440	438 ± 483	0.002

∆ Corresponds to baseline (anthropometric, body composition parameters, and resting energy expenditure) minus (anthropometric, body composition parameters, and resting energy expenditure) at 6 months.

* Significance level (two-tailed) for comparison between groups using independent Student’s test.

**Supplementary table 3:** Clinical and laboratory features of patients according to the presence of the minor alleles of each analyzed polymorphisms

Polymorphism	*PGC-1*〈 *rs8192678*		p-value	*FNDC5 rs3480*		p-value	*UCP1 rs3826*		p-value
	Risk genotype (A/A-A/G)	Other type (G/G)		Risk genotype (A/G-G/G)	Other type (A/A)		Risk genotype (G/G-G/A)	Other type (A/A)	
Weight, kg	106.4 ± 28.0	91.4 ± 29.0	0.035	97.8 ± 27.0	108.2 ± 29.6	0.143	105.4 ± 31.6	91.7 ± 22.5	0.037
BMI, kg/m^2^	40.2 ± 10.6	35.8 ± 12.4	0.078	37.0 ± 9.4	42.0 ± 12.8	0.069	40.0 ± 12.6	35.7 ± 8.8	0.158
Waist circumference, cm	117.9 ± 20.4	107.5 ± 21.9	0.048	111.6 ± 19.8	119.7 ± 21.6	0.105	117.2 ± 22.7	107.5 ± 17.7	0.062
Systolic BP, mmHg	130.9 ± 20.5	128.5 ± 16.1	0.623	130.5 ± 17.3	126.6 ± 18.3	0.384	131.1 ± 20.8	127.5 ± 15.0	0.440
Diastolic BP, mmHg	80.0 ± 15.0	79.1 ± 10.3	0.790	79.2 ± 11.9	80.1 ± 15.6	0.799	81.2 ± 14.7	77.3 ± 10.1	0.234
REE, kcal/day	1946.0 ± 571.4	1868.5 ± 722.2	0.633	1908.5 ± 592.5	2002.2 ± 677.1	0.561	1982.7 ± 705.5	1784.5 ± 500.9	0.220
Fat mass, kg	49.9 ± 19.8	40.5 ± 17.6	0.070	42.7 ± 19.0	51.7 ± 17.2	0.076	47.2 ± 19.8	42.2 ± 18.1	0.351
Fat-free mass, kg	59.5 ± 18.1	55.2 ± 19.9	0.373	57.2 ± 18.6	60.0 ± 18.5	0.455	61.9 ± 21.0	50.9 ± 12.6	0.019
Fasting glucose, mmol/L	7.3 ± 2.6	7.5 ± 2.4	0.859	7.6 ± 2.7	7.1 ± 2.3	0.443	7.6 ± 2.6	7.2 ± 2.5	0.494
Glycated hemoglobin, %	6.1 ± 1.0	6.1 ± 1.3	0.747	6.0 ± 1.1	6.2 ± 1.3	0.464	6.3 ± 1.3	5.7 ± 0.7	0.028
HOMA-IR	32.8 (11.3-162.0)	19.3 (4.9-112.3)	0.201	33.0 (6.6-135.0)	32.7 (4.9-162.0)	0.950	31.7 (6.6-112.3)	31.3 (4.9-162.0)	0.860
Total cholesterol, mg/dL	180.5 ± 45.7	181.7 ± 39.6	0.914	172.8 ± 33.5	191.7 ± 49.6	0.084	180.0 ± 39.1	180.2 ± 48.1	0.984
LDL cholesterol, mg/dL	113.0 ± 38.5	112.8 ± 38.2	0.987	107.9 ± 25.4	120.5 ± 50.1	0.259	111.8 ± 33.5	113.4 ± 44.4	0.880
HDL cholesterol, mg/dL	37.7 ± 7.8	45.4 ± 12.1	0.004	39.2 ± 8.4	42.3 ± 10.8	0.209	41.1 ± 11.5	41.1 ± 8.5	0.993
Triglycerides, mg/dL	122 (18 - 342)	103 (23 - 382)	0.206	110 (18 - 348)	117 (37 - 382)	0.397	103 (23 - 382)	110 (18 - 341)	0.986
Plasma Irisin, ng/mL	9.5 ± 2.1	10.4 ± 3.1	0.295	10.0 ± 2.8	9.8 ± 2.5	0.936	9.7 ± 2.6	10.1 ± 2.7	0.703

Data are presented as means ± standard deviation or median (interquartile range).

BMI: body mass index; BP: blood pressure; HOMA-IR: homeostatic model assessment of insulin resistance; LDL: low-density lipoprotein cholesterol; HDL: high-density lipoprotein cholesterol; REE: resting energy expenditure; UCP1: uncoupling protein 1.
